# Use of Hydrogen–Rich Gas in Blast Furnace Ironmaking of V–bearing Titanomagnetite: Mass and Energy Balance Calculations

**DOI:** 10.3390/ma15176078

**Published:** 2022-09-01

**Authors:** Xudong Gao, Run Zhang, Zhixiong You, Wenzhou Yu, Jie Dang, Chenguang Bai

**Affiliations:** 1 College of Materials Science and Engineering, Chongqing University, Chongqing 400044, China; 2 Key Laboratory of Vanadium–Titanium Metallurgy and New Materials, Chongqing University, Chongqing 400044, China; 3 College of Metallurgy and Materials Engineering, Chongqing University of Science and Technology, Chongqing 401331, China

**Keywords:** hydrogen–rich gas, v–bearing titanomagnetite, blast furnace ironmaking, mass and energy balance, operation window

## Abstract

The iron and steel industry is a major CO_2_ emitter and an important subject for the implementation of carbon emission reduction goals and tasks. Due to the complex ore composition and low iron grade, vanadium–bearing titanomagnetite smelting in a blast furnace consumes more coke and emits more carbon than in an ordinary blast furnace. Injecting hydrogen–rich gas into blast furnace can not only partially replace coke, but also reduce the carbon emission. Based on the whole furnace and zonal energy and mass balance of blast furnace, the operation window of the blast furnace smelting vanadium–bearing titanomagnetite is established in this study on the premise that the thermal state of the blast furnace is basically unchanged (raceway adiabatic flame temperature and top gas temperature). The effects of different injection amounts of hydrogen–rich gases (shale gas, coke oven gas, and hydrogen) on raceway adiabatic flame temperature and top gas temperature, and the influence of blast temperature and preheating temperature of hydrogen–rich gases on operation window are calculated and analyzed. This study provides a certain theoretical reference for the follow–up practice of hydrogen–rich smelting of vanadium–bearing titanomagnetite in blast furnace.

## 1. Introduction

In 2020, China set the goals of “carbon peaking” in 2030 and “carbon neutralization” in 2060 [1]. The iron and steel industry is a major CO_2_ emitter and an important subject for the implementation of carbon emission reduction goals and tasks. It is an urgent strategic task to vigorously develop new low–carbon or carbon–free metallurgical technologies, especially for ironmaking systems. In recent years, several researches have been carried out around low–carbon ironmaking technology at home and abroad [2,3,4,5,6], the research focuses on the reduction of iron ore by replacement carbon with hydrogen and, thus, reduction of carbon emission. Although non–blast–furnace ironmaking technology can play a certain role in carbon reduction, it is not the current mainstream ironmaking process; so, the research focus should be on blast furnace ironmaking in the short term. Blast furnace ironmaking is a highly carbon–intensive process, and each ton of hot metal (THM) produced emits roughly 1.8 tons of CO_2_, most of which is consumed in the form of coke. The reducing agent and heating agent of coke can be replaced by injecting hydrogen–rich fuel (with low carbon concentration). The carbon content of shale gas and coke oven gas is about 75% and 23%, respectively, both lower than that of coke (usually 84~91%) [7]. In industry, there have been practical cases for blast furnaces to inject hydrogen–rich gas with CH_4_ as the main component, a method widely used in North America, Russia, and other regions; for example, in 2015, 13 blast furnaces in North America (with a total output of 16.6 million THM) only injected natural gas, with an average injection volume of 83 kg/THM, and 12 blast furnaces simultaneously injected natural gas and pulverized coal [8,9,10,11].

In the 1960s and 1970s, Chongqing Iron and Steel Corporation had the practical experience of injecting natural gas into the furnace, but it was abandoned because the price of natural gas was much higher than that of pulverized coal at that time and the economic benefit was low. Now, huge reserves of shale gas resources have been explored in Southwest China, and with the continuous development of mining technology, the cost continues to decline, which provides a practical basis for injecting shale gas into the blast furnace. At the same time, China has fully mastered the smelting technology of vanadium–bearing titanomagnetite in blast furnace and formed a unique technical standard and system. However, at present, the research on smelting vanadium–bearing titanomagnetite by hydrogen–rich or full hydrogen blast furnace combined with shale gas has not been explored in–depth. In order to accurately evaluate the feasibility of hydrogen–rich gas injection into a vanadium–bearing titanomagnetite blast furnace, the current study took a 1750 m^3^ blast furnace in an iron and steel plant in Panxi area as the research object and established a mathematical model of blast furnace operation window based on the energy and mass balance of blast furnace by calculating and analyzing the reasonable operation window of shale gas, coke oven gas, and hydrogen. It provides a certain theoretical reference for the follow–up practice of hydrogen–rich smelting of vanadium–bearing titanomagnetite in blast furnace.

## 2. Mathematical Model of Operating Window for Injecting Hydrogen–Rich Gas into Blast Furnace

### 2.1. Mathematical Model of Energy and Mass Balance in Blast Furnace

According to the whole furnace and zonal heat balance [12,13,14], combined with the characteristics of hydrogen–rich gas injection, the mathematical model of energy and mass balance of hydrogen–rich gas injection was established. The preconditions for establishing energy and mass balance are as follows:

(1) The types of hydrocarbon combination in shale gas are complex, but the main component is CH_4_, and the cracking process of other alkanes is similar to that of methane. Therefore, in order to simplify the calculation process, the alkane components are converted into CH_4_ in this study.
(2) CH_4_ in the hydrogen–rich gas undergoes cracking and combustion reaction at the tuyeres to generate CO and H_2_.
(3) The relative error of material balance is within 0.1%, which ensures the accuracy of solving the gas composition equation in each zone.
(4) The heat loss of the whole furnace and area is 5~8%.

The establishment method of energy and mass balance is as follows:

(1) Mass balance calculation. The iron ore consumption was obtained according to the Fe balance. Before injecting hydrogen–rich gas, the blast volume and top gas composition were measured, and the direct reduction degree could be obtained according to actual production data. Then, when hydrogen–rich gas injection was simulated, the blast volume was obtained according to the oxygen balance; the furnace hearth and top gas compositions were calculated according to the C, H, and N balances; the direct reduction degree after injecting hydrogen–rich gas could be approximately calculated by the empirical formula as follows [14]:

(1) $$r_{d}=\frac{r_{d}^{0}\times 10^{-s\lambda }\times \left(0.684+0.01\times t_{0}^{0.5}\right)}{\left(0.96+4\varphi \right)}$$

(2) Heat balance calculation. The heat balance of the whole furnace was calculated based on the Gass Law, without considering the actual reaction process in the blast furnace, and with the material feeding state as the starting point and the output state as the end point; the zonal heat balance was based on the actual reaction process, taking into account the main energy sources and heat consumption in the blast furnace.

The thermal effects of all chemical reactions are considered in the heat balance calculation of blast furnace, which is based on the database data of FactSage 8.1 software and related literatures. The thermal effects of different chemical reactions are different at different temperatures, among which some major chemical reactions and their thermal effects are as follows:

(2) $$\mathrm{FeO}+C=\mathrm{Fe}+\mathrm{CO}\;\;\;\;\;\;q_{Fed}=-a1\times r_{d}\times 1000\times w\left[\mathrm{Fe}\right]$$

(3) $${\mathrm{SiO}}_{2}+2C=\mathrm{Si}+2\mathrm{CO}\;\;\;q_{Si}=-a2\times 1000\times w\left[\mathrm{Si}\right]$$

(4) $${\mathrm{TiO}}_{2}+2C=\mathrm{Ti}+2\mathrm{CO}\;\;\;q_{Ti}=-a3\times 1000\times w\left[\mathrm{Ti}\right]$$

(5) $$V_{2}O_{5}+5C=2V+5\mathrm{CO}\;\;\;q_{V}=-a4\times 1000\times w\left[V\right]$$

(6) $$\mathrm{FeO}+H_{2}={\mathrm{Fe}+H}_{2}O\;\;\;\;\;\;q_{FeH}=-a5\times r_{H}\times 1000\times w\left[\mathrm{Fe}\right]$$

(7) $$O_{2}+2C=2\mathrm{CO}\;\;\;\;q_{C}=a5\times w{\left(C\right)}_{tuyere}$$

(8) $$O_{2}{+2\mathrm{CH}}_{4}=2\mathrm{CO}+2H_{2}\;\;\;q_{CH_{4}}=a6\times w{\left({\mathrm{CH}}_{4}\right)}_{tuyere}$$

(9) $$\mathrm{FeO}+\mathrm{CO}={\mathrm{Fe}+\mathrm{CO}}_{2}\;\;\;\;\;\;\;\;q_{FeCO}=a7\times \left(1-r_{d}-r_{H}\right)\times 1000\times w\left[\mathrm{Fe}\right]$$

### 2.2. Thermodynamics Analysis of Reduction of Vanadium and Titanium Oxides

**Figure 1:**
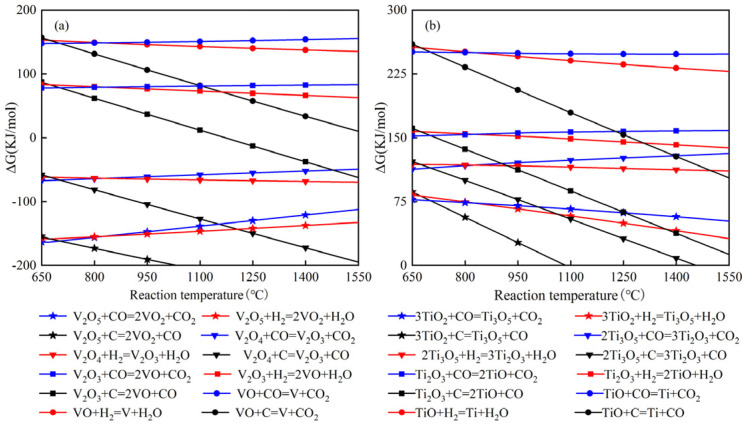
Gibbs free energy of standard reaction for reduction of vanadium oxide and titanium oxide: (**a**) vanadium oxide; (**b**) titanium oxide.

Considering that vanadium–bearing titanomagnetite contains a lot of titanium oxides and vanadium oxides, it is necessary to research the reduction of titanium oxides and vanadium oxides in the process of smelting vanadium–bearing titanomagnetite by hydrogen–rich or full hydrogen blast furnace combined with shale gas. Figure 1 depicts the Gibbs’ free energy change as a function of temperature in the reduction of V_2_O_5_ and TiO_2_ with different reducing agents (CO, H_2_, and C). It can be found that the reduction of V_2_O_5_ and TiO_2_ is carried out in stages: V_2_O_5_→V_2_O_4_→V_2_O_3_→VO→V; TiO_2_ →Ti_3_O_5_→Ti_2_O_3_→TiO→Ti. Compared with CO and H_2_, vanadium oxide and titanium oxide can be easily reduced by carbon at high temperature under standard conditions. Affected by the complex conditions of blast furnace hearth, the contents of vanadium and titanium in hot metal are 0.31% and 0.19%, respectively, according to the actual smelting data of vanadium titanium blast furnace, which are mainly obtained by direct reduction.

### 2.3. Raceway Adiabatic Flame Temperature

The raceway adiabatic flame temperature (RAFT) was calculated by assuming that the combustion products are CO, H_2_, and N_2_ only [15], considering the reaction of the blast air and injectants with carbon from coke at 1550 °C and the effects of ash–forming oxides, and the physical heat contained in the fuel and blast can be transferred to the combustion products after deducting the heat consumption in the combustion zone. With the in–depth study of physical and chemical processes in blast furnace, the RAFT calculation model [16,17,18,19] has been continuously modified and improved, and the following points were also considered based on the previous RAFT calculation: the influence of temperature on heat capacity of raw fuel, blast, and hydrogen–rich gas; the heat consumption of heating up, melting, and SiO_2_ gasification of ash after combustion; the sensible heat and reaction heat of pulverized coal and hydrogen–rich gas.

According to the injection characteristics of hydrogen–rich blast furnace, the raceway adiabatic flame temperature (RAFT) was modified from two aspects: firstly, the decomposition heat of methane was calculated by using FactSage 8.1 software; secondly, the combustion rate of shale gas was obtained by simulating the carbon balance of blast furnace.

RAFT calculation formula is

(10) $$\mathrm{RAFT}=\frac{q_{C}+q_{meth}^{r}+q_{shale}^{s}+q_{blast}^{s}+q_{coke}^{s}+q_{coal}^{s}-q_{SiO}^{r}-q_{ash}^{m}}{Q_{g}^{H}+\bar{C_{SiO}}\times V_{SiO}+\bar{C_{coke}}\times n_{coke}+\bar{C_{coal}}\times n_{coal}}$$

### 2.4. Top Gas Temperature

The top gas temperature (TGT) with certain heat loss conditions was calculated based on the zonal heat balance [14]. The influence of injecting hydrogen–rich gas and direct reduction degree on the top gas temperature was considered in the calculation, which was reflected in the items $q_{hgas}$, $q_{i}$, and $V_{tgas}$. In addition, the effects of temperature and gas composition on heat capacity ($C_{tgas}$) were also considered.

The calculation formula is as follows:

(11) $$\mathrm{TGT}=\frac{q_{hgas}+q_{i}+q_{b}^{s}-q_{p}^{c}-q_{b}^{c}-Q_{loss}^{l}}{C_{tgas}\times V_{tgas}}$$

In the middle and lower part of the blast furnace, the reducing ability of carbon is much greater than that of CO and H_2_ due to the higher temperature (gas temperature greater than 1000 °C and burden temperature greater than 950 °C, based on literature data), suggesting that the reduction reaction in this high temperature area is mainly direct reduction [14,20]. Therefore, since the direct reduction degree will directly affect the amount of gas in the high temperature area, the direct reduction enthalpy is considered in $q_{hgas}$. The calculation formula of each term of the TGT equation is as follows:

(12) $$q_{hgas}=c_{hgas}\times V_{hgas}\times T_{hgas}$$

(13) $$q_{i}=q_{co}+q_{H_{2}}$$

(14) $$q_{b}^{s}=c_{ore}\times ore\times T_{ore}+c_{coke}\times coke\times T_{coke}$$

(15) $$q_{p}^{c}=q_{H_{2}O}+q_{dust}$$

(16) $$q_{b}^{c}=q_{coke}+q_{ore}$$

It is worth noting that the individual heat in these equations varies with the type and injection rate of hydrogen–rich gas. Based on the energy mass balance model and TGT calculation model, a series of calculations (under the condition that the thermal state of the blast furnace is kept stable) can be used to obtain the relevant parameter values with injecting different hydrogen–rich gases, as shown in Table A1, Table A2 and Table A3. The variation law of the top gas composition, individual heats, and direct reduction degree with the injection rate of hydrogen–rich gas is basically consistent with the calculation in the reference [14].

### 2.5. Mathematical Model of Operation Window

**Figure 2:**
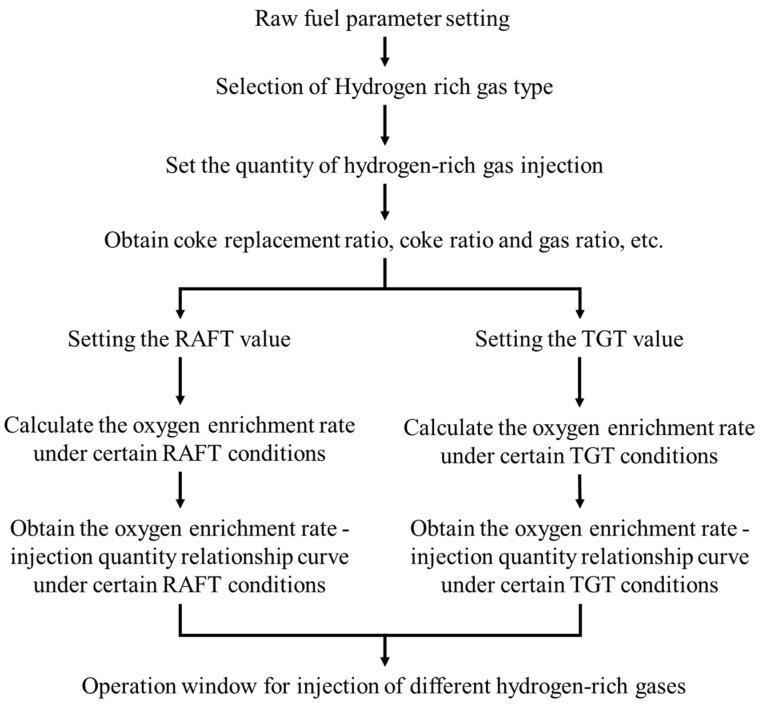
Calculation flow chart of blast furnace operation window.

When hydrogen–rich gas is injected, the amount of hearth gas increases, the RAFT decreases, and the TGT increases. The ranges of RAFT and TGT can be adjusted by increasing the blast oxygen enrichment rate. RAFT is an important parameter for the blast furnace operator to judge the thermal state of the hearth. It determines the initial gas temperature of the hearth, thus affecting the heat transfer, reduction, slagging, desulfurization, hot metal temperature, composition, etc. When hydrogen–rich fuel is injected, the RAFT becomes low, which reduces the replacement ratio, and with the increase in hydrogen–rich fuel consumption, the furnace condition may even deteriorate. Too high RAFT will also cause the initial gas volume expansion in the hearth, and a large amount of SiO volatilizes, which increases the resistance to the charge column, affects the decline of the charge, and even makes the blast furnace difficult to run and suspend. Too–high or too–low TGT is also unfavorable to the production of blast furnace. Too–high TGT will increase the heat loss in the furnace, increase the coke ratio, and shorten the service life of furnace top charging equipment and bag filter. Too–low TGT will cause the gas temperature to be lower than the dew point, further causing dust to block the filter cloth, making it lose its filtering function and greatly reducing the dust removal efficiency. Therefore, in order to discuss the operation constraints of injecting hydrogen–rich gas, the allowable injection amount of hydrogen–rich gas (Abscissa) and blast oxygen enrichment rate (Ordinate) are often used to describe the feasible operation window on the premise of maintaining the thermal state of blast furnace (reasonable RAFT and TGT) [15,21,22]. The calculation process of the operation window is shown in the Figure 2.

According to the previous study [23], the RAFT is controlled at 2050~2200 °C during pulverized coal injection and 1900 °C for natural gas injection. The lower limit of RAFT can be as low as 1704 °C when natural gas injection is recommended for some North American blast furnaces [24]. K. S. Abdel Halim et al. [25] studied the variation range of RAFT when injecting natural gas into four blast furnaces in Russia and Egypt. After injecting natural gas, RAFT showed a downward trend. For example, when SSC 1# blast furnace reached the maximum injection volume (158 m^3^/THM), the RAFT decreased by more than 200 °C. The difference in RAFT calculation methods of blast furnaces in different iron and steel plants lead to the different blast parameters, and the difference of RAFT can reach 150 °C [26]. Therefore, according to the on–site production parameters of a 1750 m^3^ blast furnace smelting vanadium–bearing titanomagnetite in Panxi, China, the RAFT is calculated to be 2150~2250 °C; in combination with the charge properties, slag characteristics, top equipment, and dust removal restrictions, this study sets the operation windows of TGT in the range 110~180 °C [27] and the RAFT in the ranges of 2000~2100 °C and 2100~2200 °C.

## 3. Calculation Conditions

Due to the large amount of calculation data of energy and mass balance, we only list the main parameters used for calculation. According to the 1750 m^3^ blast furnace, the main production parameters under normal generation conditions (only PCI) are shown in Table A4 and the composition of mixed ore is shown in Table A5. The compositions of hydrogen–rich gas (shale gas, coke oven gas, and hydrogen), coke, and PCI for calculation are shown in Table A6, Table A7 and Table A8, respectively. The composition of hot metal and slag are shown in Table A9 and Table A10, respectively. Meanwhile, the calorific value of various fuels can be obtained based on the given composition of coke, coal, and hydrogen–rich gas, as shown in Table A11 [28,29].

## 4. Calculation Results and Analysis

Blast furnace smelting is a complex and high–temperature reaction process of gas–liquid–solid countercurrent. Replacing coke with hydrogen–rich gas will change the fuel structure of blast furnace smelting, which will have a great impact on the blast furnace smelting. In order to determine the feasibility of injection of hydrogen–rich gas into blast furnace with smelting vanadium–bearing titanomagnetite, the influence of three types of injected hydrogen–rich gases (shale gas, coke oven gas, and hydrogen) on the blast furnace smelting parameters was studied.

Blast furnace smelting is a complex and high–temperature reaction process of gas–liquid–solid countercurrent. Replacing coke with hydrogen–rich gas will change the fuel structure of blast furnace smelting, which will have a great impact on blast furnace smelting. In order to determine the feasibility of injection of hydrogen–rich gas into blast furnace with smelting vanadium–bearing titanomagnetite, the influence of three types of injected hydrogen–rich gases (shale gas, coke oven gas, and hydrogen) on the blast furnace smelting parameters was studied.

**Figure 3:**
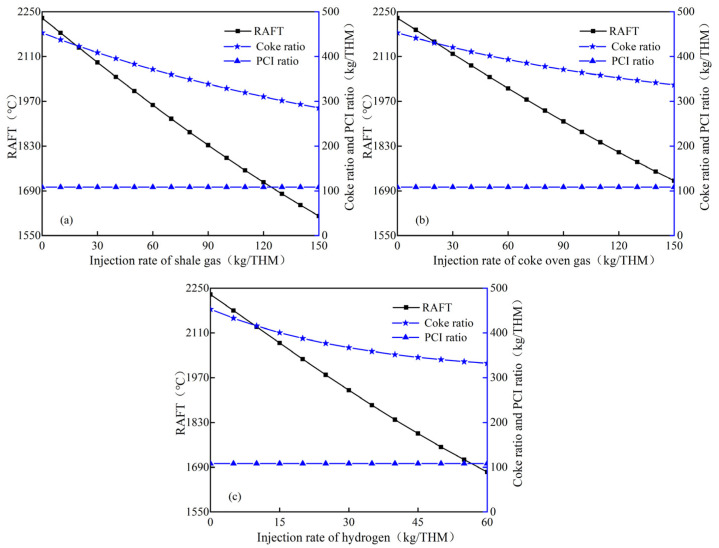
Influence of hydrogen–rich gas injection quantity on the RAFT: (**a**) shale gas; (**b**) coke oven gas; (**c**) hydrogen.

Figure 3 shows the variations of raceway adiabatic flame temperature and coke ratio after injecting hydrogen–rich gas with fixed PCI (108 kg/THM). When only pulverized coal is injected, the RAFT is 2230.3 °C. As each of the three hydrogen–rich gases replaces part of the coke and starts injection, the RAFT decreases at a slower rate with the increase in the injection quantity. However, the effect of different hydrogen–rich gases’ injection on the RAFT is obviously different. When the shale gas is injected (Figure 3a), the RAFT decreases at a rate of 4.7~3.4 °C/kg with the increase in injection quantity; the RAFT also drops to 2000 °C with the injection quantity of shale gas reaching 50.4 kg/THM. Figure 3b shows that injecting coke oven gas will decrease the RAFT by 3.7~2.9 °C/kg, while the RAFT drops to 2000 °C with an injection quantity of 62.8 kg/THM. Figure 3c shows that the decrease rate of RAFT is 10.2~7 °C /kg when pure hydrogen is injected into the furnace, and it only needs 22.8 kg/THM of injection for a RAFT of 2000 °C. This indicates that the decrease rate of the RAFT is the fastest when injecting hydrogen, while the decrease rate of the RAFT with injecting coke oven gas is the slowest. In addition, it can be also found that the coke ratio decreases at a gradually slower rate with the uniform increase in three hydrogen–rich gases injection quantity. It means that the substitution capacity of the three hydrogen–rich gases for coke gradually decreases with the increase in injection quantity, which may explain why the decreasing rate of the RAFT gradually slows down.

**Figure 4:**
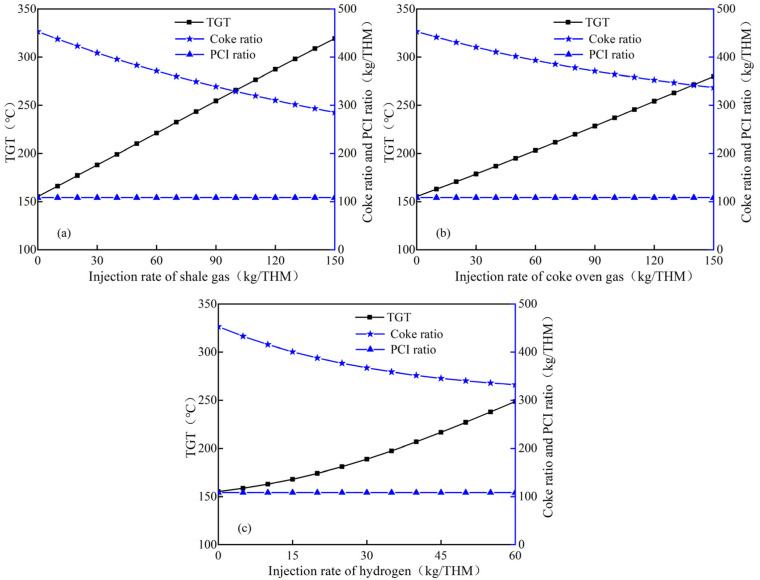
Influence of hydrogen–rich gas injection quantity on TGT: (**a**) shale gas; (**b**) coke oven gas; (**c**) hydrogen.

Figure 4 shows the variations of top gas temperature (TGT) and coke ratio after injecting hydrogen–rich gas with fixed PCI (108 kg/THM). It can be found that the initial TGT is 155.1 °C when only pulverized coal is injected. With the increase in hydrogen–rich gases injection quantity, the TGT shows an upward trend, while the increasing rate of the TGT is different with injecting different hydrogen–rich gases. As shown in Figure 4a, the increasing rate of TGT is about 1.1 °C/kg as shale gas injection quantity increases, while it is 0.8~0.9 °C/kg with coke oven gas injection (Figure 4b). This means that the injection of both hydrogen–rich gases can steadily increase the TGT. However, Figure 4c shows that the increase rate of the TGT with pure hydrogen injection varies from 0.7 to 2.2 °C/kg, which is not as stable as the injection of the other two hydrogen–rich gases. This may be caused by the increase in heat income per ton of iron in the blast furnace, which is due to the rapid decline in the substitution capacity of unit hydrogen injection quantity for coke.

Through previous calculations, it has been found that injecting hydrogen–rich gas will decrease the RAFT and increase the TGT. In order to keep the RAFT and TGT within a reasonable range, it is necessary to adjust the blast injection rate and the oxygen enrichment rate in the blast, which is called the operating window (the functional relationship between the oxygen enrichment rate and the blast injection rate). Considering that the operating window constrained by the RAFT and TGT is also affected by the blast temperature and preheating temperature of injected hydrogen–rich gases in actual production, the influence of different blast temperatures and preheating temperatures on the operating window for hydrogen–rich gas injection is also simulated.

**Figure 5:**
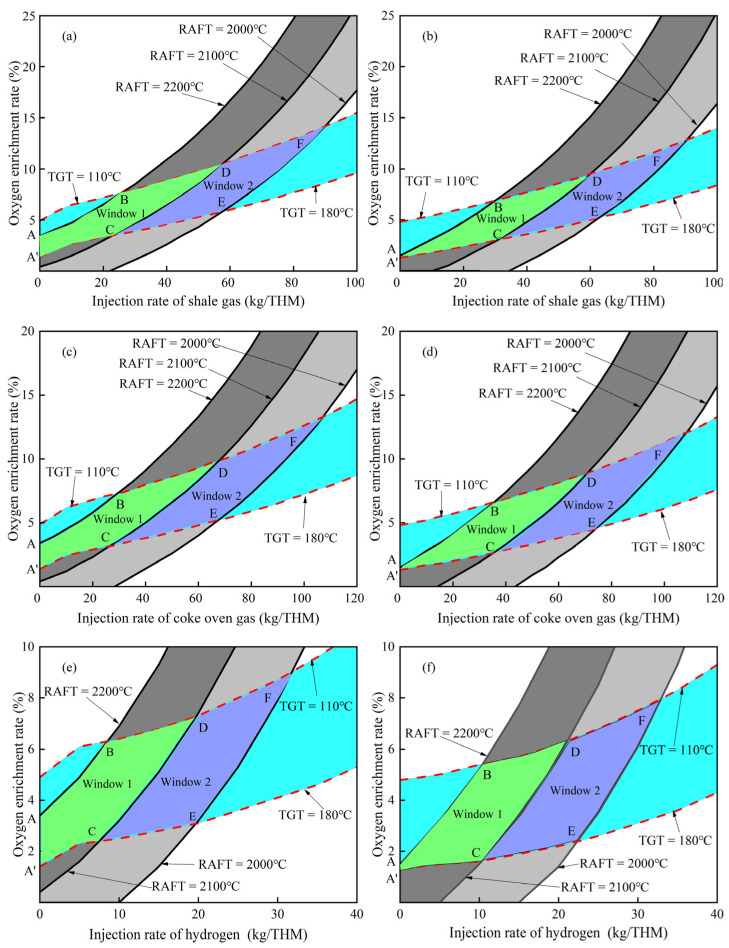
Influence of blast temperature on operation window: (**a**,**c**,**e**)—1100 °C; (**b**,**d**,**f**)—1200 °C.

Figure 5 shows the calculated operating window for three hydrogen–rich gases at two blast temperatures (at constant preheating temperature of hydrogen–rich gases of 25 °C). The constraint ranges of the TGT and RAFT are 110~180 °C and 2000~2200 °C, respectively. The difference between window 1 and window 2 is the constraint range on RAFT: window 1 is 2100~2200 °C and window 2 is 2000~2100 °C. It can be found that all the operating windows for three hydrogen–rich gases shift to the lower right with the increase in blast temperature, while the shape and area of the operation window have not changed significantly. This indicates that increasing blast temperature at the same injection rate is beneficial to reduce the upper and lower limits of oxygen enrichment rate, while the relative adjustment range of oxygen enrichment rate and injection rate will not change. As the blast temperature increases from 1100 °C to 1200 °C, the injection rate ranges of shale gas, coke oven gas, and hydrogen in their respective integrated operating windows (the constraint range of the RAFT is 2000~2200 °C) change from 0~90 kg/THM, 0~107.2 kg/THM, 0~31.8 kg/THM to 0~90.4 kg/THM, 0~108.3 kg/THM, 0~32.9 kg/THM, respectively, while the oxygen enrichment rates corresponding to their maximum injection rate change from 14.2%, 13.3%, 9% to 12.9%, 12.1%, 8%, respectively. When the injection rate of shale gas, coke oven gas, and hydrogen is 20 kg/THM, the oxygen enrichment rate ranges corresponding to their injection rate change from 3.3~7.2%, 2.9~6.8%, 3.1~7.3% to 2.4~6.1%, 2~5.7%, 2.2~6.2%, respectively. It shows that the increase in blast temperature can slightly increase the injection rate range of the operating window, but can reduce the oxygen enrichment rate of hydrogen–rich gases. In addition, it can be seen from Figure 5 that the calculated operating window for hydrogen injection has the smallest variation range for blast injection rate and oxygen enrichment rate compared with the other two hydrogen–rich gases. This means that injecting hydrogen has the lowest fault tolerance and more stringent operation requirements.

**Figure 6:**
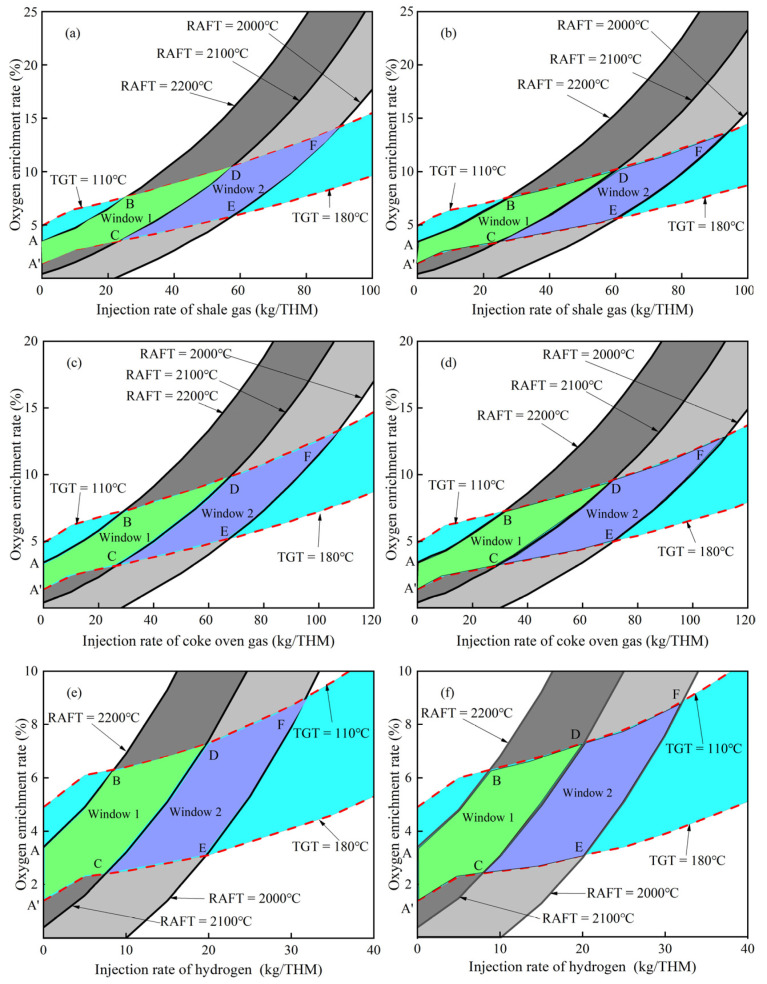
Influence of hydrogen–rich gas temperature on operation window: (**a**,**c**,**e**)—25 °C; (**b**,**d**,**f**)—600 °C. The uppercase letters (A–F) in the figure represent the vertices of the operation window.

Figure 6 shows the calculated operating window for three hydrogen–rich gases at different preheating temperatures (at constant blast temperature of 1100 °C). It can be found that when the gas injected is shale gas (Figure 6a,b) and coke oven gas (Figure 6c,d), the increase in preheating temperature will also cause the operating window to shift slightly to the lower right, demonstrating that increasing preheating temperature at the same injection rate can only slightly reduce the upper and lower limits of oxygen enrichment rate. Preheating hydrogen (Figure 6e,f) has little effect on the operating window, just a slight shift to the right. As the preheating temperature increases from 25 °C to 600 °C, the injection rate ranges of shale gas, coke oven gas, and hydrogen in their respective integrated operating windows change from 0~90 kg/THM, 0~107.2 kg/THM, 0~31.8 kg/THM to 0~94 kg/THM, 0~112.5 kg/THM, 0~32.1 kg/THM, respectively, while the oxygen enrichment rates corresponding to their maximum injection rate change from 14.2%, 13.3%, 9% to 13.7%, 13%, 8.9%, respectively. When the injection rate of shale gas, coke oven gas, and hydrogen is 20 kg/THM, the oxygen enrichment rate ranges corresponding to their injection rate change from 3.3~7.2%, 2.9~6.8%, 3.1~7.3% to 3.1~7%, 2.8~6.7%, 3.1~7.3%, respectively. This shows that the increase in preheating temperature can obviously increase the injection rate range of the operating window, but has little effect on the oxygen enrichment rate. By comparing the operation windows in the literature, it can be found that the calculated results are basically consistent with the literature [14,26].

In addition, although different hydrogen–rich gas compositions can obtain different operation windows, the operation windows obtained by theoretical calculation can be approximately seen as a parallelogram, which means that too low or too high an injection rate of hydrogen–rich gas will reduce the adjustment range of oxygen enrichment rate, thus reducing the fault tolerance rate of blast furnace operation. However, due to the lack of actual production parameters, it is difficult to make a comparison to determine the best range of injection rates for different hydrogen–rich gases.

## 5. Conclusions

The composition of injected hydrogen–rich gas has an impact on RAFT and TGT; different hydrogen–rich gas compositions can obtain different operation windows. Based on the production data of a blast furnace smelting vanadium–bearing titanomagnetite, the energy and mass balance calculation was carried out, and the operation windows of the blast furnace with hydrogen–rich gases injection were determined (the top gas temperature (TGT) was 110–180 °C, and the raceway adiabatic flame temperature (RAFT) was 2000–2200 °C). When the blast temperature increases from 1100 °C to 1200 °C, the theoretical injection quantities of shale gas, coke oven gas, and hydrogen change from 0~90 kg/THM, 0~107.2 kg/THM, 0~31.8 kg/THM to 0~90.4 kg/THM, 0~108.3 kg/THM, 0~32.9 kg/THM, respectively, and the corresponding oxygen enrichment rates change from 1.4~14.2%, 1.4~13.3%, 1.4~9% to 1.3~12.9%, 1.3~12.1%, 1.3~8%, respectively. The theoretical calculation results show that the increase in blast temperature can significantly reduce the oxygen enrichment rate of hydrogen–rich gas, while the increase in preheating temperature can significantly increase the injection quantities. Injection hydrogen–rich gas can greatly reduce the use of coke, thereby reducing greenhouse gas emissions.

## Data Availability

The data presented in this study are available on request from the corresponding author.

## Abbreviation

$q_{c}$	Heat from incomplete combustion of carbon
$q_{meth}^{r}$	Combustion heat release of CH_4_ injected into hydrogen–rich gas
$q_{blast}^{s}$	Sensible heat brought in by blast
$q_{coke}^{s}$	Sensible heat brought in by coke
$q_{coal}^{s}$	Sensible heat brought in by PCI
$q_{shale}^{s}$	Sensible heat brought in by shale gas
$q_{SiO}^{r}$	Formation heat consumption of SiO
$q_{ash}^{m}$	Heat consumption of ash melting in coke and PCI
$Q_{g}$	Heat carried away by gas in high temperature zone
$V_{CO}^{H},V_{H_{2}}^{H},V_{N_{2}}^{H},V_{SiO}^{H}$	Volumes of CO, H_2_, N_2_, and SiO in hearth gas
$\bar{C_{CO}^{H}},\bar{C_{H_{2}}^{H}},\bar{C_{N_{2}}^{H}},\bar{C_{SiO}^{H}}$	Average specific heat capacities of CO, H_2_, N_2_, and SiO in hearth gas
$n_{coke},n_{coal}$	Molar quantity of coke and PCI
$q_{hgas}$	Heat brought in by coal gas in high temperature area
$q_{i}$	Indirect reduction exothermic of H_2_ and CO
$q_{b}^{s}$	Sensible heat of furnace charge (coke and ore)
$q_{p}^{c}$	Heat consumption of water evaporation and dust
$q_{H_{2}O}$	Heat consumption of water volatilization
$q_{dust}$	Heat carried away by dust
$C_{tgas},V_{tgas},T_{tgas}$	Heat capacity, volume, and temperature of top gas
$q_{co},q_{H_{2}}$	Thermal effect of CO and H_2_ reduction
$c_{ore},ore,T_{ore}$	Heat capacity, consumption, and temperature of ore entering the furnace
$c_{coke},coke,T_{coke}$	Heat capacity, consumption, and temperature of coke ratio
$C_{hgas},V_{hgas},T_{hgas}$	Heat capacity, volume, and temperature of high–temperature gas
$r_{d}^{0}$	Direct reduction degree of reference state
$t_{0}\;$	Blast temperature in reference state
φ	Blowing humidity
s	Injection rate of hydrogen–rich gas
λ	Coefficient of injecting hydrogen–rich gas
$a_{i}$	Coefficient of thermal effect of different chemical reactions
$q_{b}^{c}$	Heat consumption of charge during heating up
$Q_{loss}^{l}$	Heat loss in low temperature zone

## Appendix A

**Table A1:** Relevant parameter values in the calculation of TGT (injection of shale gas).

Injection Rate (kg/THM)	$r_{d}$	TGT (°C)	Individual Heats (MJ/THM)					Top Gas Compositions (vol.%)			
			$q_{hgas}$	$q_{i}$	$q_{b}^{s}$	$q_{b}^{c}$	$q_{p}^{c}$	CO	CO_2_	H_2_	N_2_
0	0.481	172.0	2470.7	171.8	27.8	2071.3	47.3	26.2	18.9	2.3	52.6
10	0.452	183.6	2489.2	159.2	27.5	2041.5	46.9	25.5	18.8	3.1	52.6
20	0.424	195.3	2511.6	145.9	27.1	2013.4	46.6	25.0	18.7	4.0	52.3
30	0.399	206.9	2536.6	132.1	26.8	1986.8	46.3	24.5	18.5	4.8	52.2
40	0.374	218.5	2564.9	118.0	26.6	1961.6	46.0	24.1	18.2	5.6	52.1
50	0.352	230.0	2595.8	103.8	26.3	1937.8	45.8	23.7	17.9	6.5	51.9
60	0.33	241.5	2629.3	89.5	26.0	1915.3	45.5	23.4	17.6	7.3	51.7
70	0.31	252.9	2665.0	75.4	25.8	1893.9	45.3	23.2	17.3	8.1	51.4
80	0.291	264.2	2702.8	61.4	25.6	1873.6	45.1	22.9	16.9	8.9	51.3
90	0.274	275.5	2742.5	47.7	25.3	1854.2	44.8	22.7	16.5	9.7	51.1
100	0.257	286.7	2784.0	34.3	25.1	1835.8	44.6	22.6	16.1	10.6	50.7

**Table A2:** Relevant parameter values in the calculation of TGT (injection of coke oven gas).

Injection Rate (kg/THM)	$r_{d}$	TGT (°C)	Individual Heats (MJ/THM)					Top Gas Compositions (vol.%)			
			$q_{hgas}$	$q_{i}$	$q_{b}^{s}$	$q_{b}^{c}$	$q_{p}^{c}$	CO	CO_2_	H_2_	N_2_
0	0.481	171.9	2470.7	171.8	27.8	2071.3	47.3	26.2	18.9	2.3	52.6
10	0.454	180.4	2485.7	159.4	27.5	2047.6	47.0	25.6	18.8	3.1	52.5
20	0.428	188.9	2503.8	146.3	27.3	2025.4	46.8	25.1	18.7	3.9	52.3
30	0.404	197.5	2524.7	132.9	27.1	2004.6	46.6	24.7	18.5	4.8	52
40	0.381	206.1	2548.3	119.2	26.9	1985.1	46.4	24.4	18.3	5.6	51.7
50	0.359	214.7	2574.2	105.3	26.7	1966.7	46.2	24.1	18.0	6.4	51.5
60	0.339	223.4	2602.3	91.5	26.5	1949.5	46.0	23.8	17.7	7.2	51.3
70	0.319	232.1	2632.5	77.8	26.3	1933.3	45.8	23.6	17.4	8.1	50.9
80	0.301	240.8	2664.5	64.2	26.2	1918.0	45.7	23.4	17.1	8.9	50.6
90	0.284	249.5	2698.3	50.8	26.0	1903.6	45.5	23.2	16.7	9.7	50.4
100	0.268	258.3	2733.7	37.8	25.9	1890.1	45.4	23.1	16.4	10.5	50

**Table A3:** Relevant parameter values in the calculation of TGT (injection of hydrogen).

Injection Rate (kg/THM)	$r_{d}$	TGT (°C)	Individual Heats (MJ/THM)					Top Gas Compositions (vol.%)			
			$q_{hgas}$	$q_{i}$	$q_{b}^{s}$	$q_{b}^{c}$	$q_{p}^{c}$	CO	CO_2_	H_2_	N_2_
0	0.481	172.0	2470.7	171.8	27.8	2071.3	47.3	26.2	18.9	2.3	52.6
5	0.428	177.2	2471.2	143.0	27.4	2029.1	46.8	24.9	18.9	4.1	52.1
10	0.382	182.8	2483.3	111.7	27.0	1992.5	46.5	23.9	18.6	5.9	51.6
15	0.34	189.0	2505.1	79.2	26.7	1960.6	46.2	23.1	18.1	7.7	51.1
20	0.303	196.0	2535.1	46.7	26.4	1932.8	45.9	22.5	17.5	9.5	50.5
25	0.269	203.6	2572.1	14.7	26.2	1908.5	45.6	22.0	16.8	11.4	49.8
30	0.24	212.0	2615.1	−16.1	26.0	1887.4	45.4	21.6	16.1	13.3	49

**Table A4:** Production parameters of blast furnace for calculation.

Coke Ratio (kg/THM)	PCI Ratio (kg/THM)	Ore Consumption (t/THM)	Utilization Factor (THM/(m^3^·Day))	Oxygen Enrichment Rate (%)	Blast Temperature (°C)	Basicity
453	108	1.859	2.3	2.5	1200	1.27

**Table A5:** Mixed ore composition for calculation (mass%).

FeO	Fe_2_O_3_	CaO	SiO_2_	MgO	FeS	Al_2_O_3_	MnO	P_2_O_5_	TiO_2_	V_2_O_5_	Total	H_2_O
7.78	63.25	10.15	6.45	1.93	0.12	1.77	0.17	0.15	7.51	0.72	100.0	0.32

**Table A6:** Hydrogen–rich gas composition for calculation (vol.%).

Gas Type	CH_4_	CO_2_	N_2_	H_2_	CO	O_2_	H_2_O	H_2_S	Total
Shale gas	96.98	1.20	1.00	—	—	—	0.80	0.02	100.0
Coke oven gas	26.48	2.00	4.99	60.04	5.99	0.50	—	—	100.0
Hydrogen	—	—	—	100.00	—	—	—	—	100.0

**Table A7:** Coke composition for calculation (mass%).

Fixed Carbon	Ash Content					Volatile Matter and Organic Matter						Total	H_2_O
	SiO_2_	Al_2_O_3_	FeO	CaO	MgO	CO	CO_2_	CH_4_	H_2_	N_2_	S		
85.86	5.95	3.97	0.85	0.8	0.71	0.27	0.31	0.09	0.22	0.31	0.66	100.0	0.75

**Table A8:** PCI composition for calculation (mass%).

Fixed Carbon	Ash Content					Volatile Matter and Organic Matter				Total	H_2_O
	SiO_2_	Al_2_O_3_	FeO	CaO	MgO	S	H_2_	N_2_	O_2_		
75.85	7.18	4.07	0.74	0.98	0.29	0.67	4.54	2	3.68	100.0	1.1

**Table A9:** Hot metal composition for calculation (mass%).

[Fe]	[Si]	[Mn]	[P]	[S]	[Ti]	[V]	[C]	Total
94.07	0.18	0.15	0.12	0.08	0.19	0.31	4.90	100.0

**Table A10:** Slag composition for calculation (mass%).

CaO	SiO_2_	MgO	Al_2_O_3_	MnO	FeO	TiO_2_	V_2_O_5_	S/2	Total
32.55	26.03	6.62	9.69	0.22	0.31	23.01	1.28	0.29	100.0

**Table A11:** Calorific value of various fuels.

* Coke (MJ/kg)	* PCI (MJ/kg)	^△^ Shale Gas (MJ/m^3^)	^△^ Coke Oven Gas (MJ/m^3^)	^△^ Hydrogen (MJ/m^3^)
30.55	29.31	38.44	18.85	12.66

* Calorific value of coke and PCI is calculated according to the calculation formula in the reference [28,29]. ^△^ Calorific value of shale gas, coke oven gas, hydrogen is calculated based on the data in Table A6 by using FactSage 8.1 software (Thermfact/CRCT, Montreal, Canada; GTT–Technologies, Ahern, Germany).
